# A Propensity Score Matching Study Between Microwave Ablation and Radiofrequency Ablation in Terms of Safety and Efficacy for Benign Thyroid Nodules Treatment

**DOI:** 10.3389/fendo.2021.584972

**Published:** 2021-03-09

**Authors:** Hao Jin, Jinrui Fan, Ligong Lu, Min Cui

**Affiliations:** ^1^ The Second Department of General Surgery, Zhuhai People’s Hospital (Zhuhai Hospital Affiliated with Jinan University), Zhuhai, China; ^2^ Zhuhai People’s Hospital (Zhuhai Hospital Affiliated with Jinan University), Zhuhai, China

**Keywords:** microwave ablation, radiofrequency ablation, quality of life, complication, recurrence

## Abstract

**Background:**

Large benign thyroid nodules often lead to cosmetic problems and compression on trachea. Thermal ablation is an effective method for benign thyroid nodules treatment. Among all the thermal ablation techniques, microwave and radiofrequency are frequently used energy sources. However, treatment outcomes of the two ablation types have not been compared in detail. Therefore, we conducted this study aiming for comparing the safety and efficacy of the two ablation techniques in benign thyroid nodules treatment.

**Methods:**

Information was retrospectively collected from patients with benign thyroid nodules, who received radiofrequency ablation or microwave ablation between January 1, 2018, and December 31, 2019, in a main hospital in South China. Patients were divided into microwave ablation group and radiofrequency ablation group according to the techniques applied. A propensity score matching was performed to balance the baseline indexes between the two groups. We also recorded and analyzed the operative variables including operative duration, intraoperative blood loss, hospitalization time, and overall costs. Postoperative quality of life, volume reduction rates, and complication rates were routinely evaluated during the follow-up by asking patients to fulfil questionnaires at the 1^st^, 3^rd^, 6^th^, 12^th^, and 18^th^ postoperative month.

**Results:**

A total of 943 patients receiving microwave ablation or radiofrequency ablation in the years of 2018 and 2019 met our inclusion criteria. After 1:1 propensity score matching, 289 pairs of patients were matched. There was no significant difference between the two groups in operative duration, intraoperative blood loss, hospitalization time, overall cost, quality of life scores, complication rates or volume reduction rates.

**Conclusion:**

There was no significant difference between microwave and radiofrequency ablation in terms of safety and efficacy. Both of the two techniques` are ideal therapeutic methods in benign thyroid nodules treatment.

**Registration number:**

ChiCTR2000034764.

## Introduction

The morbidity of benign thyroid nodules (BTNs) increases every year in the world wide (1). Even though BTNs do not metastasize, large BTNs may lead to compression on trachea and esophagus (2), and also cause cosmetic problems. Conventional thyroidectomy is routinely applied in BTNs patients, but often leaves scars on patients’ necks. Endoscopic thyroidectomy transfers the scar to some more concealed positions, but requires establishing subcutaneous tunnels and leads to extra trauma to patients (3). When bilateral thyroid lobes were removed during thyroidectomy, patients after surgery often need to receive levothyroxine replacement therapy, while long-term levothyroxine may bring about side effects including osteoporosis, particularly in middle-aged females (4).

Luckily, the above discussed disadvantages of conventional thyroidectomy could be offset by the application of thermal ablation. Thyroid thermal ablation is able to destroy BTNs *via* thermal energy by inserting an electrode into them (5). No obvious scar would be left on the neck and thyroid lobes could be preserved. In this way, no oral levothyroxine is necessary. Commonly used thermal ablation techniques include microwave ablation (MWA), radiofrequency ablation (RFA), high intensity focused ultrasound (HIFU) ablation, and laser ablation (LA) (6), among which MWA and RFA are the most frequently applied. Both methods have been reported to possess high safety and efficacy (7, 8). But the operative variables and complication rates of the two ablation methods have not been compared in detail. In evaluating the efficacy of a therapy method, postoperative quality of life (QoL) is a significant factor needing to be taken into consideration, while no study has been reported comparing MWA to RFA in terms of QoL currently (9). Therefore, we conducted this study to compare MWA to RFA regarding operative variables (operative duration, intraoperative blood loss, hospitalization time, and overall cost), postoperative QoL, and incidence rates of complications (horseness, skin burn, incision infection, and postoperative hemorrhage). By this detailed comparison, we aim to verify a more ideal therapy method for patients with BTNs.

## Materials and Methods

### Ethical Approval and Consent to Participate

All procedures performed in studies involving human participants were in accordance with the ethical standards of the institutional review boards of Zhuhai People’s Hospital (Zhuhai, China) and the 1964 Helsinki Declaration and its later amendments or comparable ethical standards. This study had been approved by the Institutional Review Board of Zhuhai People’s Hospital (Zhuhai Hospital Affiliated with Jinan University) [No,2018-(03)]. Written consents were exempted since this is a retrospective study.

### Sample Size

The study was powered to detect a difference in VRR of 8%. The outcome data on 260 participants were required for 90% power, with a 5% two-sided significance level assumed. Two hundred eighty-six participants need to be inflated to so as to allow for 10% attrition in the primary outcome.

### Patients

Patients receiving MWA or RFA for the treatment of BTNs in the hospital between January 1, 2018, and December 31, 2019, were recruited into our study.

#### Inclusion criteria:

Patients were included if they meet all of the following criteria at the same time: a) ages between 18 to 80 years old; b) without respiratory, circulatory, or metabolic diseases; c) normal coagulation functions; d) normal hepatic and kidney functions; e) not allergic to the local anesthesia drugs; f) twice fine needle aspiration cytology (FNAC) confirming the benign nature of the nodules; g) maximum diameter of BTNs ≥ 2 cm; h) BTNs were more than 2 mm apart from the thyroid capsule; i) patients with no more than five BTNs (For patients with multiple BTNs, twice FNAC were respectively performed for each BTN); j) patients not being addicted to smoking or drinking; k) patients fulfilled the QoL questionnaire before the ablation and at the 1^st^, 3^rd^, 6^th^, 12^th^, and 18^th^ postoperative month.

#### Exclusion criteria:

Patients would be excluded if they meet at least one of the following criteria: a) ages < 18 or > 80 years old; b) patients with basic function disorders, including disorders in respiratory, circulatory, or metabolic systems; c) patients with abnormal coagulation functions; d) patients with abnormal liver or kidney functions; e) patients who are allergic to the local anesthesia drugs; f) patients without twice FNAC results confirming the benign nature of nodules; g) maximum diameters of BTNs < 2 cm; h) BTNs were no more than 2 mm apart from thyroid capsule; i) Patients with more than five BTNs; j) patients being addicted to smoking or drinking; k) patients who had not fulfilled the QoL questionnaire before the ablation or at the 1^st^, 3^rd^, 6^th^, 12^th^, and 18^th^ postopertive month.

### *Propensity* Score Matching Procedure

The recruited patients were assigned to the MWA or RFA group according to the different energy applied in their ablation therapy.

To balance the two groups regarding indexes including age, gender, number of BTNs, average volume of BTNs, Body mass index (BMI), and preoperative QoL scores, a 1:1 propensity score matching was performed. The procedure was completed by SPSS 22.0 (IBM Co. Ltd. Armonk, New York, USA). And the logistic regression model was constructed using the variables including age, gender, BMI, BTNs number, average BTNs volume, energy delivered and QoL score as independent variables (**Table 1**).

**Table 1:** The baseline indicators of the two groups before propensity score matching.

Variables	Microwave group (n = 532)	Radiofrequency group (n = 411)	P value
Age (year)	48 (35, 88)	52 (39, 91)	0.038
BMI (Kg/m^2^)	22.8(20.2,23.9)	21.2(19.8,23.9)	0.018
Number of BTNs	5 (1, 10)	3 (1,8)	0.012
Average volume of BTNs (ml)	8.1 (5.6, 18.9)	9.8 (5.1,19.8)	0.008
Preoperative QoL scores	289 (181, 410)	258 (151, 410)	0.006
Energy delivered	11.56 (1.87, 25.68)	16.72 (2.61, 28.57)	0.013

Patients in Microwave group were treated with microwave ablation; Patients in Radiofrequency group were treated with radiofrequency ablation; Data were reported as median (min–max).

### Ablation Procedure

Ablation procedure was performed in the hospital by physicians with 20 years of experience. All of these operative physicians had been trained in the same thyroid ablation training program and were all qualified by certificates. Therefore, the operators in the hospital were considered to be equally skilled in thyroid ablation.

### MWA

**Figure 1:**
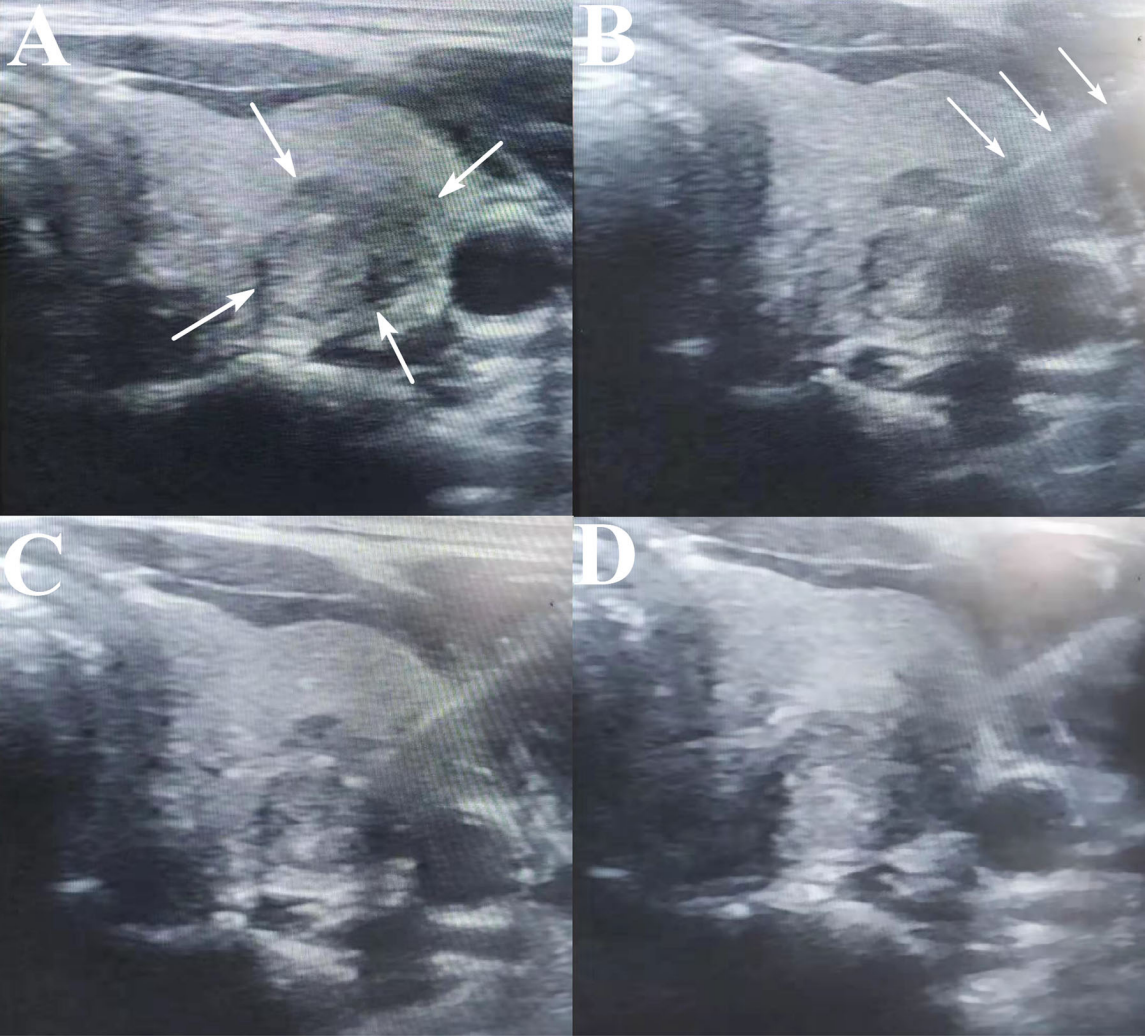
The images of a 46 year-old female undergoing MWA. **(A)** The BTN before ablation: the arrows indicate BTN. **(B, C)**. During the ablation procedure: the arrows indicate the ablation electrode. **(D)** The BTN after ablation procedure.

A tumor microwave therapy system (MTI-5DT, Changcheng Co.Ltd. Nanjing, Jiangsu Province, China) was applied in this study. For MWA procedure, operators inserted a specific microwave electrode, which has a microwave ejector on the tip, into the BTN subcutaneously. The microwave energy from the electrode would rapidly rotate the surrounding molecules. Friction among the molecules would heat up, solidify and dehydrate the BTN tissues, leading to BTN necrosis. Water-cooling equipment in the system prevents the temperature from rising too fast, so that tissue surrounding BTN would not be damaged. The dot-to-dot technique was applied so that each part of BTN could be damaged (**Figure 1**).

### RFA

As the thyroid is small and superficially located, thyroid-dedicated electrodes for RFA, which are shorter and thinner than the electrodes used in other organs, are necessary. And precise treatment was allowed by small active tips. Different active tips (5, 7, 10 mm) were applied according to the diameter of nodules. Radiofrequency therapy equipment (S-1500, Maide Co.Ltd. Shanghai, China) was applied in this study. RFA equipment and operative procedures were similar to that of MWA. We concluded that some techniques should be applied in the ablation procedure. Firstly, at the beginning of ablation, the electrode should be positioned at the bottom of BTNs, and then moved layer by layer until the top layer of BTNs has been ablated. This procedure was also known as the “moving shot” technique. Secondly, a trans-isthmic approach should be applied, which refers to the RFA electrode needs to be inserted *via* the isthmus. This approach allows the electrode position to remain stable even when a patient talks or coughs. And the normal isthmic parenchyma between the electrode and the target nodule could prevent the leakage of hot ablated fluid to the perithyroidal area. The electrode needs to be positioned still for a time of 10–15 s, so that the ablated tissues could be completely destroyed. Last but not least, for some cases with BTNs close to the vital vessels, a barrier between BTN and the significant structures (trachea and carotid artery) should be established by injecting normal saline infusion to thyroid tissues. And since recurrent laryngeal nerves (RLNs) are also located in the area between the trachea and thyroid glands, the association between the electrode, target nodule, and RLNs needs to be constantly monitored in order to prevent possible thermal injury during the ablation procedure (10, 11).

### *Operative* Variables *and* Intraoperative Complications

During ablation procedure, the operative duration, intraoperative blood loss and intraoperative complications were recorded and analyzed. Intraoperative blood loss was calculated by weighing the gauze wiping the blood. The calculation formula was: intraoperative blood loss (mL) = (weight of gauze after wiping the blood − weight of gauze before wiping the blood) (g)/1.06 g/mL (density of blood). Intraoperative complications of patients including hemorrhage, skin burn and pain were recorded.

### Hospitalization Time and Overall Costs

Hospitalization time and overall costs were recorded after patients were discharged from hospital. In these two hospitals, ablation operation was conducted in the inpatient department rather than in the outpatient department due to the medical insurance policy and the limited equipment in the outpatient department. The essential preoperative examinations such as ultrasound (US) on the neck, X-ray on the chest, coagulation function test, thyroid function test, etc. would be completed in the out-patient department. Patients often receive ablation operation at the same day to hospitalization and stay overnight in the in-patient department for necessary observation. Then they would be discharged from hospital at the next day. Hospitalization time was calculated from the hour when patients registered in inpatient department to the hour when patients were discharged from hospitals. So the hospitalization time of most patients is between 1.0 and 1.5 days. Overall costs have been conversed to US dollars (USDs) during analysis. Thyroid thermal ablation often costs little, but the disposable ablation electrode accounts for a large part of the overall cost.

### Follow-Up

**Figure 2:**
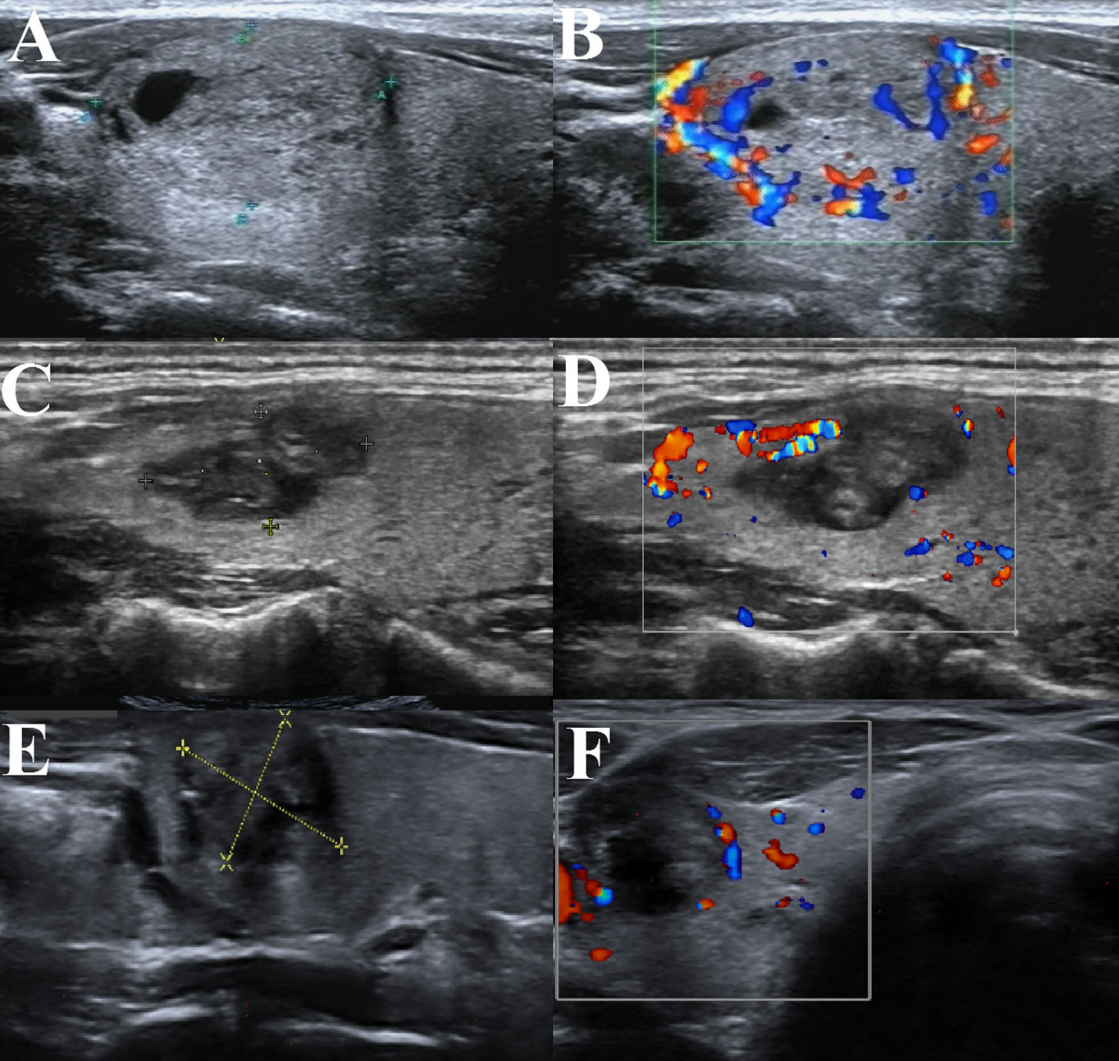
BTN volume reduction of a patient in our hospital undergoing thyroid thermal ablation. **(A)** BTN before thyroid ablation; **(B)** Perfusion signal of the BTN before ablation; **(C)** The same BTN at 3 months after ablation; **(D)** Perfusion signal of the BTN at 3 months after ablation; **(E)** The same BTN at 6 months after ablation; **(F)** Perfusion signal of the BTN at 6 months after ablation.

Patients returned to hospital for US examination at the 1^st^, 3^rd^, 6^th^, 12^th^, and 18^th^ postoperative month. By US examination, the volume reduction rate (VRR) was calculated (**Figure 2**). The postoperative complications including hematoma, horseness and cough after drinking would also be checked by physicians.

The VRR was calculated according to the volume of BTN measured at each follow-up. At the US examination during each follow-up, the diameters of BTN were measured. Volume of a BTN was calculated according to the following formula: Volume (ml) = 0.479 × π × a× b× c (cm) (a stands for the largest diameter of BTN; b and c stands for the other diameters of BTN). VRR was calculated according to the following formula: VRR = (volume measured in previous follow-up − volume measured in this follow-up)/volume measured in previous follow-up × 100%.

Patients would fulfil a thyroid QoL questionnaire respectively before ablation and at the 1^st^, 3^rd^, 6^th^, 12^th^, and 18^th^ postoperative month to evaluate patients’ satisfaction. The QoL questionnaire was made by Korean Thyroid Association and we translated it into Chinese (12). The QoL questionnaire was composed by 41 items and could be divided into 4 parts, evaluating the total spiritual well-being, total psychological well-being, total social well-being, and total physical well-being respectively. Each item has an item ranging from 0 to 10, resulting in a total score of the questionnaire ranging from 0 to 410. The average QoL score of questionnaires at the 1^st^, 3^rd^, 6^th^, 12^th^, and 18^th^ postoperative month would be calculated. In this way, the two techniques could be compared in terms of preoperative QoL.

Patients were also told to return to hospital whenever they had discomforts.

### Statistical Analysis

We had discussed with the data monitoring committee, which is independent, and then made this decision. The council met on December 01, 2018 and raised concerns about our original analysis plan’s method appropriateness considering the outcome data’ distribution in the interim report.

Appropriate descriptive statistics was applied to summarize data at all-time points. Percentages and frequencies were used for categorical data; and median (minimum, maximum) was used for continuous data. We used Mann-Whitney test to compare continuous variables and used ordinal logistic regression to compare categorical variables. Categories used in ordinal logistic regression models for skewed continuous variables were recorded in detail. From binary logistic regression, the odds ratios (ORs) was examined for the data binary splits. By this examination and Brant test with a significance threshold of P < 0.01, the proportional odds assumption was checked as appropriate. Other outcome was analyzed with generalized linear models which have appropriate link functions for the outcome distribution.

We adjusted the analysis of all the outcomes for a relevant baseline score and for the minimization variables group. Some subgroup and sensitivity analyses were performed for the outcome, including a linear regression analysis for QoL, an operation-restricted analysis performed by consultants, a per protocol analysis, analyses using multiple imputation, binary logistic regression analyses for each split of the data. For all measures of effect, 95% CIs are provided. Exploratory subgroup analyses were also performed for the outcomes, mainly for the following prespecified variables: number of BTNs ≤ 3 or > 3. A treatment by subgroup interaction term was included in the corresponding ordinal logistic regression model, so that these tests could be performed. We used bivariate correlation test so that the relationship between VRR and baseline volume could be evaluated. We used the Fischer exact test to compare small percentages. We applied a power analysis with a one-sided α level set as 8% for the VRR, so that we could estimate whether the resultant sample size had sufficient magnitude to compare RFA to MWA or not.

We used SPSS 22.0 (IBM Co. Ltd. Vermont, New York, USA) to conduct the statistical analyses. The significance level was defined as *p* value of less than 0.05.

## Results

We looked at 532 patients treated with RFA and 411 treated with MWA and finally, 92 pairs were precisely matched and 197 pairs were fuzzily matched. The baseline indicators were balanced between the two groups (**Table 2**).

**Table 2:** The baseline indicators of the two groups after propensity score matching.

Variables	Microwave group (n = 532)	Radiofrequency group (n = 411)	P value
Age (year)	49 (38, 89)	49 (38, 90)	0.53
BMI (kg/m^2^)	22.1(20.2,23.9)	22.2(20.2,23.9)	0.87
Number of BTNs	4 (1, 8)	4 (1,8)	0.90
Average volume of BTNs (ml)	9.2 (5.6, 18.9)	9.2 (5.6,18.9)	0.81
Preoperative QoL scores	271 (181, 410)	271 (181, 410)	0.75
Energy delivered (KJ) 13.17 (2.96, 25,12)		13.26 (2.72, 25.78)	0.89

Patients in Microwave group were treated with microwave ablation; Patients in Radiofrequency group were treated with radiofrequency ablation; Data were reported as median (min–max).

### *Operative* Variables, Hospitalization Time and Overall Costs

Operative duration, hospitalization time, intraoperative blood loss, overall costs were compared between the RFA group and MWA group. The results showed that there was no significant difference between the two groups in operative duration, intraoperative blood loss, hospitalization time, and overall cost. Even though we found that MWA required less shot, significant difference did not exist in operative duration between the two groups (**Table 3**). The electrode costs almost 1,600 USDs, accounting for over 60% of the overall cost. Operation fee costs only about 10% of the overall cost, indicating a rather reasonable charge standard of medical care in China’s public hospitals. Some medicine to stop bleeding accounts for another 10% of the overall cost. Hospitalization expenses and nursing expenses also accounts for about 10% of the overall cost.

**Table 3:** Operative duration, intraoperative blood loss, hospitalization time, and overall costs in RFA and MWA groups.

Operative variables	Microwave group (n=289)	Radiofrequency group (n=289)	P value
Operative duration (min)	29.2 (26.0, 32.4)	28.9 (27.6, 30.2)	0.32
Intraoperative blood loss (ml)	1.1 (0.9, 1.3)	1.2 (1.1, 1.3)	0.28
Hospitalization time (day)	1.3 (1.2, 1.4)	1.2 (1.0, 1.4)	0.72
Overall costs	2567.1(2545.9,2588.3)	2571.4(2539.1,2603.7)	0.71
Operation cost	308.6(300.1, 317.8)	309.3(299.2, 319.6)	
Care cost	359.2 (341.2,377.2)	352.7 (340.1,364.8)	
Drug cost	314.2 (302.4, 326.8)	326.2 (312.5,340.6)	
Other cost (electrode cost, etc)	1585.1 (1562.8, 1603.1)	1583.2 (1561.3, 1605.8)	

In Microwave group, patients were treated with microwave ablation; In Radiofrequency group, patients were treated with radiofrequency ablation. Except for P values, variables were presented as mean (minimum, maximum).

### Complications

Complications rates were calculated and obtained through operative procedure and follow-up. The complication incidence rate was similar between the two groups. The most common complication in the two groups was skin burn and pain. Two patients in the MWA group and three patients in the RFA group reported skin burn and pain. But skin burn was not serious and pain was relived from the five patients at the next day of operation without oral supplementation of painkillers. Horseness was also common in patients of the two groups. One patient in MWA group and two patients in RFA group reported horseness. However, voice of these patients returned to normal during follow-up. One patient recovered at the first day after ablation. Two patients recovered within a month. Two patients (one in MWA group and one in RFA group) reported cough after drinking, but both of them recovered within a month. Hematoma was seen in only one patient, which may be caused by incomplete electrocoagulation. By compression, the hematoma disappeared at the third day after ablation. Other complications were not seen (**Table 4**).

**Table 4:** Complications including horseness, skin burn and pain, hematoma, and cough after drinking in MWA group and RFA group.

Complications	Microwave group (n=289)	Radiofrequency group (n = 289)	P value
Total	1.73% (5/289)	2.06%(6/289)	0.73
Hemorrhage and hematoma	0.34% (1/289)	0	0.12
Skin burn and pain	0.69% (2/289)	1.03% (3/289)	0.37
Mild	0.35% (1/289)	0.35% (1/289)	
Moderate	0.35% (1/289)	0.35% (1/289)	
Severe	0	0.35% (1/289)	
Horseness	0.35% (1/289)	0.67% (2/289)	0.72
Cough after drinking	0.35% (1/289)	0.35% (1/289)	0.49

In Microwave group, patients were treated with microwave ablation; In Radiofrequency group, patients were treated with radiofrequency ablation; Data were reported as % (n/N.).

### Postoperative QoL Scores

Postoperative QoL scores were not significantly different between RFA group and MWA group. There was also no significant difference between the two groups in terms of total QoL scores at the 1^st^, 3^rd^, 6^th^, 12^th^, and 18^th^ postoperaive month. Moreover, there was no significant difference between the two groups in the scores of total physical well-being, total psychological well-being, total spiritual well-being, and total social well-being. In RFA group, patients with total scores >400 accounted for 58% (167/289), total scores of 300–400 accounted for 19% (55/289), total scores of 200–300 accounted for 13% (38/289), and total scores < 200 accounted for 10% (29/289). 13% (38/289) of the patients reported total scores of 410 (the maximum total score). In MWA group, patients with total scores >400 accounted for 59% (170/289), 300–400 for 20% (58/289), 200–300 for 12% (35/289), < 200 for 9% (26/289). 12% (34/289) of the patients reported the maximum total score of 410 (**Table 5**).

**Table 5:** Patients postoperative QoL score in MWA group and RFA group.

Postoperative QoL score	Microwave group (n=289)	Radiofrequency group (n=289)	Adjusted effect size	P value
Average postoperative QoL scores	341.57 (334.25, 348.89)	343.91 (335.20, 352.62)	1.02 (0.78 to 1.52)	0.32
1^st^ month QoL score	291.38 (284.1, 298.66)	293.46 (286.65, 300.27)	0.81 (0.72 to 1.03)	0.59
3^rd^ month QoL score	310.59 (301.83, 319.35)	312.49 (304.93, 320.05)	0.71 (0.56 to 0.91)	0.68
6^th^ month QoL score	337.86 (329.93, 345.79)	341.58 (332.67, 350.46)	1.09 (0.71 to 1.25)	0.81
12^th^ month QoL score	352.19 (340.27, 364.11)	353.78 (340.96, 366.59)	0.82 (0.56 to 1.26)	0.38
18^th^ month QoL score	372.89 (360.08, 385.70)	373.19 (360.68, 385.70)	0.32 (0.13 to 0.58)	0.67
Overall QoL scores				
Total physical well-being	93.2 (85.88, 100.52)	92.9 (86.32, 99.48)	1.52 (1.32 to 1.89)	0.78
Total psychological well-being	91.7 (81.92, 101.48)	93.9 (83.34, 104.46)	1.38 (1.09 to 3.56)	0.39
Total spiritual well-being	91.32 (79.4, 103.04)	92.97 (79.78, 106.16)	1.52 (1.32 to 1.86)	0.38
Total social well-being	72.56 (63.44, 81.68)	73.91 (62.34, 85.48)	1.08 (1.03 to 1.95)	0.24
Proportions of QoL score > 400	59% (170/289)	58% (167/289)	0.38 (0.31 to 0.49)	0.38
Proportions of QoL score=410	12% (34/289)	13% (38/289)	0.59 (0.21 to 0.89)	0.42
Proportions of QoL score 300–400	20% (58/289)	19% (55/289)	0.41 (0.18 to 0.97)	0.50
Proportions of QoL score 200–300	12% (35/289)	13% (38/289)	0.95 (0.52 to 1.39)	0.41
Proportions of QoL < 200	9% (26/289)	10% (29/289).	0.28 (0.11 to 0.59)	0.17

In Microwave group, patients were treated with microwave ablation; In Radiofrequency group, patients were treated with radiofrequency ablation; Data were represented with % (n/N) or mean (minimum, maximum); Odds ratio from ordinal logistic regression or binary logistic regression; Adjusted difference were used in percentages; Difference in means from linear regression.

### VRR

VRR at the 1^st^, 3^rd^, 6^th^, 12^th^, and 18^th^ month was respectively obtained and there was no significant difference between the two groups (**Table 6**). The outcome of recurrence has been defined as a volume increase > 50% as compared to the previous smallest volume recorded by US (13). We found only 1 recurrence among 578 patients. The patient was a 45 year-old female and the BTN had a preliminary volume of 10.28 ml. The patient received secondary ablation treatment. After 6 months, VRR of this BTN reached 67.2% and no recurrence was found.

**Table 6:** VRR (%) in microwave and radiofrequency group.

Follow up	Microwave group (n=289)	Radiofrequency group (n=289)	P value
1^st^ month	15.3 (8.2, 22.4)	15.4 (8.2, 22.6)	0.32
3^rd^ month	47.9 (37.7, 58.1)	48.2 (36.9, 59.5)	0.72
6^th^ month	67.8 (59.9, 75.7)	68.1 (60.0, 76.2)	0.91
12^th^ month	79.3 (76.1, 82.5)	80.1 (78.3, 81.9)	0.56
18^th^ month	91.7 (88.5, 94.9)	89.2 (84.4, 94.0)	0.58

Data were presented as mean (min, max) variations.

## Discussion

Both RFA and MWA destroy BTN using thermal energy, and the necrotic BTN tissues would be absorbed gradually by the surrounding thyroid tissues. Some physicians were worried about the radiation risk of radiofrequency, although there was no verified evidence. MWA was applied since 2012 by Feng et al. (14). Compared to RFA, MWA equipment has been adjusted so that it could be easier to use. Microwave coagulation was developed in the early 1980s during hepatic resection in order to achieve hemostasis (15). For some time, the thermal effect of MWA was suspicious about, since RFA ranges from 300 MHz to 300 GHz, whereas MWA generators currently allow only two frequency spectrums, namely 915 MHz and 2.45 GHz. However, currently MWA has substantially changed the field of thermal ablation in interventional oncology. MWA devices function within the RF spectrum and can technically be defined as a subset of RFA (16). So MWA and RFA, which is the better approach for thyroid thermal ablation? Our study compared RFA to MWA in terms of efficacy and safety, promising to provide an evidence for physicians to choose the most appropriate method.

Both methods belong to thermal ablation, and no significant difference exists between the two methods in operative variables, including operative duration, intraoperative blood loss, hospitalization. There was also no significant difference between the two groups in terms of overall costs. In fact, the most expensive part of this therapy method is the ablation electrode, which is of one-time use. The cost of ablation electrodes used in these two methods are similar, about 1,600 USDs. The cost of such an electrode is not fully covered by the Residents Medical Assurance in most hospitals of China. So the cost of the ablation electrode increases economic burdens on patients and society, and limited the generalization of this technique. A reduction of the cost of electrodes would significantly reduce the overall cost of thyroid thermal ablation treatment. Besides, thyroid thermal ablation is associated with mini-invasion, rapid recovery, and local anesthesia, and this technique could be completed at the out-patient department. But in many hospitals in China, thyroid thermal ablation is only performed in the in-patient departments owing to the limited equipment in the our-patient departments, and this situation could be further improved in the future.

In our previous study (17), VRR in the MWA group at the 12^th^ postoperative month could reach 79.6%. But the long-term outcome of thermal ablation is still to be verified. Hence we performed a follow-up of 18 months in this study. Similar to our previous study, VRR in this study is also satisfying. And the VRR at the 18^th^ postoperative month reached about 90% in both groups. So we have a confidence that the VRR could reach 100% at the 24^th^ postoperative month. And a further study with a longer follow-up is necessary. Another satisfying result of this study is that the recurrence rate after ablation is extremely low. We found only one recurrence among 578 patients. However, the follow-up in our study is not adequate to register regrowths, so further study would be performed in the future.

In our study, no significant difference exists between the RFA and MWA group in terms of VRR and complication rates. Consistent with the treatment outcomes, there was no significant difference in QoL scores between the two groups. These results are easy to be interpreted, since patients QoL largely depends on treatment efficacy and safety. In our previous study (18), patients’ postoperative satisfaction and QoL are much higher in the ablation group than that in the conventional thyroidectomy group, showing a better psychological well-being in thyroid thermal ablation patients. Those patients allocated to thyroid thermal ablation group reported higher quality of life values than those allocated to conventional thyroidectomy, possibly representing quicker recovery and better cosmetic results. In our study, both groups take advantages over conventional thyroidectomy since thyroid thermal ablation (both MWA and RFA) was noted to be minimal invasiveness and good cosmetic outcomes.

Similar to our study, research by Solis-Gutierrez D et al. (19), Korkusuz Y et al. (20) and Park HS et al. (21) also confirm that no significant difference exists between RFA and MWA in complication rates. Research by Yue W et al. (22) showed that there was no significant difference between RFA and MWA in VRRs. The study by Vorländer C et al. (23) also reported no significant difference in the operative duration and VRR, but MWA requires less shots to treat the whole nodule, which was also similar to our study. However, no existing study compares the postoperative QoL or intraoperative blood loss between MWA and RFA.

However, some research reported different results. For instance, Hu K et al. (24) reported a higher VRR at the 6^th^ and 12^th^ postoperative month in the RFA group compared to MWA group. The different equipment and operative ability of operators in these studies may contribute to this discrepancy. The small sample size of both studies may also lead to the discrepancy.

At the same time, our study has some limitations, such as a small sample size and a short follow-up. And the operative complications and overall cost need to be further analyzed. We would also like to take these shortages into consideration, and the efficacy and safety of the two techniques could be further verified in our future research.

## Conclusions

As far as we know, this is the first study comparing the efficacy and safety of MWA and RFA in terms of operative variables (operative duration, intraoperative blood loss, hospitalization time, overall costs), operative complications (hemorrhage and hematoma, skin burn and pain, horseness, and cough after drinking), VRR, and QoL scores. We concluded that there was no significant difference between the two techniques, and both of the two techniques are safe and effective methods for benign thyroid nodules treatment.

## Data Availability Statement

The raw data supporting the conclusions of this article will be made available by the authors, without undue reservation.

## Ethics Statement

The studies involving human participants were reviewed and approved by Zhuhai People’s Hospital (Zhuhai Hospital Affiliated with Jinan University). Written informed consent for participation was not required for this study in accordance with the national legislation and the institutional requirements. Written informed consent was obtained from the individual(s) for the publication of any potentially identifiable images or data included in this article.

## Author Contributions

LL and MC proposed the study. LL, HJ and MC conducted investigation and statistical analysis. HJ and JF drafted the manuscript. LL and MC are the guarantors. All authors contributed to the article and approved the submitted version. 

## Conflict of Interest

The authors declare that the research was conducted in the absence of any commercial or financial relationships that could be construed as a potential conflict of interest.

## Abbreviations

MWA, microwave ablation; RFA, radiofrequency ablation; BTN, benign thyroid nodule; FNAC, fine needle aspiration cytology; BMI, body mass index; QoL, quality of life; LA, laser ablation; HIFU, high intensity focused ultrasound; USD, US dollars; US, ultrasound; VRR, volume reduction rate.

## References

[1] Cavallo A Johnson DN White MG Siddiqui S Antic T Mathew M. Thyroid Nodule Size at Ultrasound as a Predictor of Malignancy and Final Pathologic Size. Thyroid (2017) 27:641–50. 10.1089/thy.2016.0336 28052718

[2] Cipriani NA White MG Angelos P Grogan RH. Large Cytologically Benign Thyroid Nodules Do Not Have High Rates of Malignancy or False-Negative Rates and Clinical Observation Should be Considered: A Meta-Analysis. Thyroid (2018) 28:1595–608. 10.1089/thy.2018.0221 30280990

[3] Cho J Park Y Baek Y Sung K. Single-incision endoscopic thyroidectomy for papillary thyroid cancer: A pilot study. Int J Surg (2017) 43:1–6. 10.1016/j.ijsu.2017.05.030 28502882

[4] Pasandideh R Hosseini SM Veghari G Hezarkhani S. The Effects of 8 Weeks of Levothyroxine Replacement Treatment on Metabolic and Anthropometric Indices of Insulin Resistance in Hypothyroid Patients. Endocr Metab Immune (2020) 20:745–52. 10.2174/1871530319666191105123005 31702509

[5] Chen J Cao J Qiu F Huang P. The Efficacy and The Safety of Ultrasound-guided Ablation Therapy for Treating Papillary Thyroid Microcarcinoma. J Cancer (2019) 10:5272–82. 10.7150/jca.36289 PMC6775625 31602278

[6] Cho SJ Baek JH Chung SR Choi YJ Lee JH. Thermal Ablation for Small Papillary Thyroid Cancer: A Systematic Review. Thyroid (2019) 29:1774–83. 10.1089/thy.2019.0377 31739738

[7] Cui T Jin C Jiao D Teng D Sui G. Safety and efficacy of microwave ablation for benign thyroid nodules and papillary thyroid microcarcinomas: A systematic review and meta-analysis. Eur J Radiol (2019) 118:58–64. 10.1016/j.ejrad.2019.06.027 31439259

[8] Chung SR Suh CH Baek JH Park HS Choi YJ Lee JH. Safety of radiofrequency ablation of benign thyroid nodules and recurrent thyroid cancers: a systematic review and meta-analysis. Int J Hyperthermia (2017) 33:920–30. 10.1080/02656736.2017.1337936 28565997

[9] Lan Y Luo Y Zhang M Jin Z Xiao J Yan L. Quality of Life in Papillary Thyroid Microcarcinoma Patients Undergoing Radiofrequency Ablation or Surgery: A Comparative Study. Front Endocrinol (2020) 11:249. 10.3389/fendo.2020.00249 PMC7242647 32499754

[10] Papini E Monpeyssen H Frasoldati A Hegedüs L. 2020 European Thyroid Association Clinical Practice Guideline for the Use of Image-Guided Ablation in Benign Thyroid Nodules. Eur Thyroid J (2020) 9:172–85. 10.1159/000508484 PMC7445670 32903999

[11] Kim JH Baek JH Lim HK Ahn HS Baek SM Choi YJ. 2017 Thyroid Radiofrequency Ablation Guideline: Korean Society of Thyroid Radiology. Korean J Radiol (2018) 19:632–55. 10.3348/kjr.2018.19.4.632 PMC6005940 29962870

[12] Ryu CH Park B Ryu J Ryu YM Jo SA Lee YJ. Development and Evaluation of a Korean Version of a Thyroid-Specific Quality-of-Life Questionnaire Scale in Thyroid Cancer Patients. Cancer Res Treat (2018) 50:405–15. 10.4143/crt.2017.012 PMC5912148 28602058

[13] Bernardi S Giudici F Cesareo R Antonelli G Cavallaro M Deandrea M. Five-Year Results of Radiofrequency and Laser Ablation of Benign Thyroid Nodules: A Multicenter Study from the Italian Minimally Invasive Treatments of the Thyroid Group. Thyroid (2020) 30:1759–70. 10.1089/thy.2020.0202 32578498

[14] Feng B Liang P Cheng Z Yu X Yu J Han Z. Ultrasound-guided percutaneous microwave ablation of benign thyroid nodules: experimental and clinical studies. Eur J Endocrinol (2012) 166:1031–7. 10.1530/EJE-11-0966 22447813

[15] Tabuse K Katsumi M Kobayashi Y Noguchinull H Egawanull H Aoyama O. Microwave surgery: hepatectomy using a microwave tissue coagulator. World J Surg (1985) 9:136–43. 10.1007/BF01656265 3984365

[16] Vogl TJ Nour-Eldin NA Hammerstingl RM Panahi B Naguib NNN. Microwave Ablation (MWA): Basics, Technique and Results in Primary and Metastatic Liver Neoplasms. Rofo (2017) 189:1055–66. 10.1055/s-0043-117410 28834968

[17] Jin H Fan J Liao1 K He Z Li W Cui M. A propensity score matching study between ultrasound-guided percutaneous microwave ablation and conventional thyroidectomy for benign thyroid nodules treatment. Int J Hyperthermia (2018) 35:232–8. 10.1080/02656736.2018.1492028 30176761

[18] Jin H Lin W Lu L Cui M. Conventional thyroidectomy versus thyroid thermal ablation on postoperative quality of life and satisfaction for patients with benign thyroid nodules. Eur J Endocrinol (2020) 184:131–41. 10.1530/EJE-20-0562 33112273

[19] Negro R Trimboli P. Thermal ablation for benign, non-functioning thyroid nodules: A clinical review focused on outcomes, technical remarks, and comparisons with surgery. Electromagn Biol Med (2020) 39:347–55. 10.1080/15368378.2020.1809448 32799679

[20] Korkusuz Y Groner D Raczynski N Relin O Kingeter Y Grunwald F. Thermal ablation of thyroid nodules: are radiofrequency ablation, microwave ablation and high intensity focused ultrasound equally safe and effective methods? Eur Radiol (2018) 28:929–35. 10.1007/s00330-017-5039-x 28894936

[21] Park HS Baek JH Park AW. Values and limitations of the comparing thyroid radiofrequency and microwave ablation using propensity score. Endocrine (2017) 56:681–2. 10.1007/s12020-017-1302-9 28424969

[22] Yue WW Wang SR Lu F Sun LP Guo LH Zhang YL. Radiofrequency ablation vs. microwave ablation for patients with benign thyroid nodules: a propensity score matching study. Endocrine (2017) 55:485–95. 10.1007/s12020-016-1173-5 27905049

[23] Vorländer C David Kohlhase K Korkusuz Y Erbelding C Luboldt W Baser I. Comparison between microwave ablation and bipolar radiofrequency ablation in benign thyroid nodules: differences in energy transmission, duration of application and applied shots. Int J Hyperthermia (2018) 35:216–25. 10.1080/02656736.2018.1489984 30300014

[24] Hu K Wu J Dong Y Yan Z Lu Z Liu L. Comparison between ultrasound-guided percutaneous radiofrequency and microwave ablation in benign thyroid nodules. J Cancer Res Ther (2019) 15:1535–40. 10.4103/jcrt.JCRT_322_19 31939434

